# Exploring Labor Analgesia as an Alternative to Caesarean Section: A Cross‐Sectional Study on What Influences Painless Delivery Intentions Amongst Pregnant Women

**DOI:** 10.1155/prm/7659412

**Published:** 2026-03-28

**Authors:** Xue Cheng, Xiaoling Li, Jinlan Xu, Paulo Moreira

**Affiliations:** ^1^ Department of Nursing, The First Affiliated Hospital of Shandong First Medical University & Shandong Provincial Qianfoshan Hospital, Jinan, China; ^2^ International Healthcare Management Research and Development Center, The First Affiliated Hospital of Shandong First Medical University & Shandong Provincial Qianfoshan Hospital, Jinan, China; ^3^ Gestao em Saude, Atlantica Instituto Universitario, Oeiras, Portugal

**Keywords:** caesarean section, healthcare management, labor analgesia, painless delivery, self-rating scale of painless intention to deliver

## Abstract

**Background:**

In early 2025, the report by the World Health Organization indicates that caesarean section use continues to rise accounting for more than 21% of all world childbirths and predicts that C‐sections will be used in nearly 30% of all births globally by 2030, this being well above intended rate of roughly 10%–15% The rate of caesarean section in Brazil is over 50%, and in China, it is already as high as 41%, of which 31.2% are undertaken without clinical indication. Labor analgesia can reduce the rate of caesarean section by influencing women’s willingness to give birth vaginally. Yet, many pregnant women are not aware of the painless delivery alternatives.

**Objective:**

The objective of this article is to generate evidence on painless delivery intentions among pregnant women and identify influencing factors.

**Methodology:**

A total of 404 pregnant women were surveyed using the general information questionnaire and the self‐rating scale of painless intention to deliver based on the theory of planned behavior. STROBE protocol guidelines were followed.

**Results:**

Pearson correlation analysis was performed based on the scores of basic knowledge, behavioral attitude, subjective norm, and perceptual behavior control of the self‐rating scale of painless delivery intention in the planned behavior theory. The results suggest a positive correlation between basic knowledge, behavioral attitude, subjective norm, and perceptual behavior control, with correlation coefficients. Rural versus urban backgrounds and monthly household income were the two main influencing factors for the choice of painless delivery. Further correlation analysis revealed that perceived behavioral control exhibited the strongest correlation with behavioral intention in the linear regression model for total intention score, which was the largest in magnitude among all predictors, indicating it is the strongest predictor of behavioral intention toward painless childbirth.

**Conclusions:**

The results of this study suggest that there are a number of health management challenges for healthcare professionals, in general and nursing professionals, in particular. A key challenge is the ongoing need to generate international evidence on the current status of painless delivery intentions among pregnant women and identify influencing factors. This type of evidence will allow a diverse set of comparative international options for intervention based on the different influencing factors identified in this study.

**Summary**
• Problem or issue?
◦ WHO reported that caesarean section accounts for more than 21% of all world childbirths, and in China, it already reaches 41%, 31.2% being without clinical indication. Evidence on women’s knowledge concerning safe and effective alternatives is urgent.
• What is already known?
◦ Evidence suggests that labor analgesia could reduce the cesarean section rate by influencing women’s willingness to give birth vaginally. “Labor analgesia” is also known as painless delivery.
• What this paper adds?
◦ Pregnant women hold low awareness of painless delivery alternatives. The article clarifies the dimensions required for professional intervention to raise awareness and acceptance.

## 1. Introduction

The World Health Organization (WHO) has noted a global upward trend in caesarean section use, which poses unnecessary short‐ and long‐term health risks to women and newborns when performed without clinical indication. This trend is expected to continue in the coming decade, and recent WHO publications have further confirmed this ongoing challenge [1–3].

In China, the overuse of caesarean sections is particularly prominent, and labor analgesia (also known as painless delivery) has been identified as a potential strategy to reduce this rate by enhancing women’s willingness for vaginal delivery [4]. Labor analgesia encompasses pharmacological and nonpharmacological approaches [5]. Currently, no analgesic method is entirely optimal: nonpharmacological modalities are underutilized due to factors such as insufficient midwifery staff, with limited data supporting their efficacy [6]. Among pharmacological options, neuraxial block analgesia is the most widely used and reliable, achieving an analgesic effectiveness rate of over 95% and being regarded as the closest to an “ideal” solution [6]. Despite this, China’s labor analgesia rate remains below 10%, a stark contrast to the over 85% rate in developed European and American countries [8].

A key barrier to broader adoption of painless delivery is limited awareness among pregnant women, as “maternal acceptance” significantly influences its uptake [7]. To address this gap, the present study draws on the theory of planned behavior (TPB) to explore pregnant women’s intention to choose painless delivery and its influencing factors, with the goal of providing evidence for subsequent interventions to improve natural delivery rates. The TPB comprises four core components: behavioral attitude, subjective norms, perceived behavioral control, and behavioral intention. To comprehensively capture the drivers of painless delivery intention, this study supplements the TPB with a basic knowledge dimension, forming a modified theoretical model to guide the investigation.

## 2. Methods

### 2.1. Research Subjects

#### 2.1.1. Selection Criteria

Inclusion criteria: (1) Singleton pregnancy, (2) confirmed pregnancy from diagnosis to the onset of labor, (3) capable of independent understanding and voluntary cooperation with the investigation, and (4) provided informed consent.

Exclusion criteria: (1) Those with a history of mental illness, (2) those who cannot give birth through vaginal delivery as assessed by a doctor, (3) those who refuse to give birth through the vagina, (4) those for whom neuraxial anesthesia is contraindicated, and (5) those who do not agree to cooperate with the investigation.

#### 2.1.2. Sampling Method

From August to October 2020, convenience sampling was used to recruit participants. Eligible pregnant women were those undergoing prenatal examinations at the outpatient department of a tertiary hospital in Shandong Province (explicitly specifying recruitment from outpatient clinics, not wards or laboratories).

Questionnaires were administered through two channels: online via the Wenjuanxing platform and offline using paper questionnaires. The average time required to complete the questionnaire was 3–5 min. A total of 420 questionnaires were collected, of which 404 were valid, with an effective recovery rate of 96.19%. The method of averaging the missing data in the questionnaire was supplemented.

#### 2.1.3. Sample Size Determination

The sample size was calculated based on the number of independent variables from general data and the items of the self‐rating scale of painless delivery intention based on the TPB, considering the inclusion of 5–10 times the number of independent variables for surveying:

The scale contains 30 items. There are 7 variables in the general data. A 10% invalid sample rate was accounted for to address potential questionnaire ineffectiveness.

The final calculated sample size range was 204–407, which was consistent with the actual valid sample size of 404.

### 2.2. Research Instruments

Two research instruments were used in this study.

#### 2.2.1. General Information Questionnaire

Self‐developed to collect demographic and clinical‐related data, including 7 variables: age, parity, educational level, occupation, household registration type, monthly household income, and frequency of participation in prenatal school courses.

#### 2.2.2. Self‐Rating Scale of Painless Delivery Intention

The self‐compiled self‐rating scale of painless delivery intention based on the TPB is provided as Supplementary Material, with its development and validation process as follows: It was formulated through three rounds of correspondence consultations with 22 experts using the Delphi method, followed by a presurvey involving 250 pregnant women for item analysis and reliability/validity testing; exploratory factor analysis

(EFA) extracted 4 common factors (basic knowledge, behavioral attitude, subjective norm, and perceived behavioral control) with a cumulative variance contribution rate of 70.506%; confirmatory factor analysis (CFA) demonstrated good model fit (*χ* ^2^/df = 2.357, RMSEA = 0.079, CFI = 0.899); validity was confirmed by content validity (S‐CVI = 0.984, I‐CVI = 0.909–1.000), and structural validity was supported by EFA and CFA, while reliability was verified with an overall Cronbach’s *α* coefficient of 0.971 and a split‐half reliability of 0.931; the final scale consists of 4 dimensions and 30 items, adopting a 5‐point Likert scale (1 = “completely inconsistent” to 5 = “completely consistent”), specifically, 4 items for basic knowledge, 8 items for behavioral attitude, 2 items for subjective norm, and 16 items for perceived behavioral control.

### 2.3. Quality Control

#### 2.3.1. Investigation Stage

Unified training was provided to all research team members to ensure a consistent understanding of the study’s purpose, significance, methods, and questionnaire content.

Standardized guidelines were used for questionnaire administration.

Participants were selected strictly in accordance with the inclusion and exclusion criteria.

Completed questionnaires were checked on‐site immediately after submission; errors or omissions were corrected promptly.

#### 2.3.2. Statistical Stage

Dual‐person data entry was implemented to minimize data entry errors.

### 2.4. Ethical Principles

Ethical approval: This study was approved by the Ethics Committee of the First Affiliated Hospital of Shandong First Medical University (Approval no.: 02‐20240208).

Informed consent: Prior to questionnaire completion, participants were informed of the study’s purpose and significance; the survey was conducted only after obtaining their voluntary consent.

Confidentiality: All questionnaires were completed anonymously. Participant information was used exclusively for this study and not disclosed to any third party.

### 2.5. Statistical Analysis

Data entry: Excel 2016 was used for data entry.

Statistical software and methods:

SPSS 23.0 was applied for univariate analysis (*t*‐test/ANOVA), multiple linear regression analysis, and Pearson correlation analysis.

Amos 24.0 was used for CFA of the scale.

Significance level: The test level was set at *α* = 0.05.

## 3. Results

### 3.1. Current Status of Pregnant Women’s Choice of Painless Delivery

Among the 404 surveyed pregnant women, 157 (38.861%) were aged 26–30 years old, accounting for the largest proportion, which was basically consistent with the best childbearing age (25–29) years. 223 (55.198%) were primiparous mothers. 175 (43.317%) of pregnant women had a bachelor’s degree. There were more nonagricultural household registrations, with a total of 243 (60.149%). The monthly household income was the highest at 5000–10,000 yuan, with 139 people (34.406%). According to the survey on the participation of pregnant women in school courses, 299 (74.010%) pregnant women have never participated in school courses for pregnant women, which is more related to the restrictions on the number of medical visits in 2020, and the use rate of online classes for pregnant women is low in the early stage of promotion. The results of this study showed that the total score of painless delivery intention was 150 points, and the mean score was (115.827 ± 16.756) points. The full score of the basic knowledge part is 20 points, and the mean score is (14.901 ± 2.031) points. The full score of the behavioral attitude part was 40 points, and the mean score was (30.696 ± 5.280) points. The full score of the subjective normative part was 10 points, and the mean score was (7.401 ± 1.590). The full score of the perceptual behavior control part was 80 points, and the mean score was (62.829 ± 9.376) points.

### 3.2. Factor Analysis of Painless Delivery Intention Self‐Rating Form Based on TPB

One‐way analysis of the total score of the self‐rating scale of painless labor intention based on the TPB:

Table 1 shows that there are statistically significant differences in the total score of pain‐free delivery intention among pregnant women with different household registration types and monthly household income, and the score of nonagricultural household registration is higher than that of agricultural household registration.

**TABLE 1:** One‐way analysis of scale total score (*n* = 404).

Project	Group	Average value *X* <	Standard error	*F*/*t*	*p*	LSD
Age	16–20	131.800	16.400	1.046	0.406	
	21–25	116.756	17.224			
	26–30	115.841	17.291			
	31–35	114.253	16.096			
	≥ 36	118.581	15.154			

Parity	0	115.960	17.178	0.531	0.661	
	1	115.970	16.691			
	2	115.730	15.948			
	3	103.670	10.214			

Household registration type	Nonagriculture	120.040	15.465	6.507	*p* < 0.001	1 > 2
	Agriculture	109.470	16.722			

Monthly household income	< 5000	107.760	15.004	6.238	*p* < 0.001	1 < 2, 3, 4, 5; 4 > 1, 2, 3, 5
	5000–10,000	112.730	14.561			
	10,000–15,000	118.120	17.060			
	15,000–20,000	121.320	19.592			
	≥ 20,000	117.360	14.958			

Educational level	Junior college and below	131.430	15.873	1.693	0.151	
	Senior high school, technical school, polytechnic school	115.790	17.146			
	Junior college	116.000	16.913			
	Undergraduate	115.800	16.742			
	Master degree or above	114.280	16.241			

Number of times participating in pregnancy school courses	0	115.990	16.938	0.980	0.430	
	1	112.950	18.634			
	2	114.820	15.553			
	3	120.460	13.968			
	4	104.330	9.866			
	≥ 5	104.563	11.753			

Career	Civil servants	115.590	20.075	0.573	0.820	
	Medical staff	116.450	16.764			
	Laborer	122.750	16.721			
	Teacher	113.360	16.083			
	Official	115.310	17.107			
	Technician	115.680	16.810			
	Merchant	122.910	16.819			
	Farmer	108.000	15.556			
	Unemployed	113.900	17.124			
	Others	117.230	16.146			

*Note:* Hukou type: 1, nonagricultural; 2, agricultural. Monthly household income: 1, < 5000; 2, 5000–10,000; 3, 10,000–15,000; 4, 15,000–20,000; 5, ≥ 20,000.

#### 3.2.1. Descriptive Analysis

Regarding age, the participants had a minimum age of 23 and a maximum of 54, corresponding to an average of 38.50 years, with a standard deviation (SD) of 8.270 years and a coefficient of variation (CV) of 21.03%, which shows a moderate dispersion around the average.

Univariate analysis of basic knowledge of painless delivery intention self‐rating scale based on TPB:

There were statistically significant differences in the scores of basic knowledge of painless delivery among pregnant women living in urban or rural areas, education levels, and monthly family income (*p* < 0.01), and the scores of urban areas were higher than those of rural areas.

Univariate analysis of behavioral attitudes based on the self‐rating scale of painless delivery intention based on the TPB:

There were statistically significant differences in the scores of attitudes toward pain‐free childbirth among pregnant women living in rural and urban areas and monthly household income (*p* < 0.001), and the scores of urban areas were higher than those of rural areas.

Subjective normative univariate analysis of the self‐rating scale of painless delivery intention based on the TPB:

There was a statistically significant difference in the subjective normative score of painless delivery among pregnant women with different household registration types and monthly household income (*p* < 0.05), and the score of nonagricultural household registration was higher than that of agricultural household registration, the subjective normative score of different family monthly income groups was higher than that of other groups by the LSD test, the subjective normative score of the group with monthly household income < 5000 was lower than that of other groups, and the subjective normative score of the group with monthly household income of 15,000–20000 was higher than that of other groups.

Univariate analysis of perceptual behavior control based on the self‐rating scale of painless labor intention based on the TPB:

There were statistically significant differences in the perceptual behavior control scores of pain‐free delivery among pregnant women with different household registration types and monthly family income (*p* < 0.001), and the scores of nonagricultural household registration were higher than those of agricultural households.

### 3.3. Linear Regression Analysis Based on the Scores of the Self‐Rating Scale of Painless Labor Intention Based on the TPB

The statistically significant variables in univariate analysis were taken as independent variables (see Table 2 for assigning values to each variable), and the total score of intention, basic knowledge, attitude, subjective norm, and perceptual behavior control were used as dependent variables for linear regression analysis, and the linear relationship between each influencing factor and the total score and the scores of each dimension was discussed.

**TABLE 2:** Assignment of independent variables.

Project (*X*)	Value
Household registration type (*X*1)	Nonagricultural = 1, agricultural = 2
Monthly household income (*X*2)	< 5000 = 1, 5000–10,000 = 2, 10,000–15,000 = 3, 15,000–20,000 = 4, ≥ 20,000 = 5
Educational level (*X*3)	Junior college and below = 1, senior high school, technical school, polytechnic school = 2, junior college = 3, undergraduate = 4, master degree or above = 5

Linear regression of the total score of the self‐rating scale of painless delivery intention based on the TPB is shown in Table 3.

**TABLE 3:** Linear regression results of the total score of the scale.

Independent variables	Unstandardized coefficient		Standardized coefficient	*t*	*p*
	*B*	Standard error	Beta		
Constant	107.019	2.899	—	36.912	< 0.001
Household registration type	−8.180	1.400	−0.279	−5.841	< 0.001
Monthly household income	1.858	0.595	0.149	3.123	0.002

*Note: R* = 0.338; *R* ^2^ = 0.114.

Linear regression of basic knowledge scores on the self‐rating scale of painless delivery intention based on the TPB:

The basic knowledge score was taken as the dependent variable, indicating that the regression equation was statistically significant, as shown in Table 4.

**TABLE 4:** Linear regression results of basic knowledge scores.

Independent variables	Unstandardized coefficient		Standardized coefficient	*t*	*p*
	*B*	Standard error	Beta		
Constant	16.980	0.615	—	27.611	< 0.001
Household registration type	−0.882	0.208	−0.213	−4.244	< 0.001
Monthly household income	0.229	0.086	0.130	2.674	0.008
Educational level	−0.409	0.105	−0.193	−3.900	< 0.001

*Note: R* = 0.292; *R* ^2^ = 0.085.

Linear regression of behavioral attitude scores on the painless labor intention self‐rating scale based on the theory of planning behavior:

Taking the behavioral attitude score as the dependent variable and the urban/rural background and monthly household income as independent variables, the multiple linear regression equation (*α* in = 0.05, *α* out = 0.1) was introduced, and the regression equation was *Y* = 32.437 − 2720*X*1 + 0.716*X*2, and the results of ANOVA were *F* = 22.732, *p* < 0.001, indicating that the regression equation was statistically significant, as shown in Table 5.

**TABLE 5:** Linear regression results of behavioral attitude scores.

Independent variables	Unstandardized coefficient		Standardized coefficient	*t*	*p*
	*B*	Standard error	Beta		
Constant	32.437	1.073	—	30.226	< 0.001
Household registration type	−2.720	0.518	−0.252	−5.248	< 0.001
Monthly household income	0.716	0.220	0.156	3.250	0.001

*Note: R* = 0.319; *R* ^2^ = 0.102.

Linear regression of subjective normative scores on the painless labor intention self‐rating scale based on the TPB indicating that the regression equation was statistically significant, as shown in Table 6.

**TABLE 6:** Linear regression results of subjective norm scores.

Independent variables	Unstandardized coefficient		Standardized coefficient	*t*	*p*
	*B*	Standard error	Beta		
Constant	8.165	0.326	—	25.079	< 0.001
Household registration type	−0.845	0.157	−0.260	−5.371	< 0.001
Monthly household income	0.145	0.067	0.105	2.164	0.031

*Note: R* = 0.297; *R* ^2^ = 0.088.

Linear regression of perceptual behavior control scores on the painless labor intention self‐rating scale based on the theory of planning behavior indicating that the regression equation was statistically significant, as shown in Table 7.

**TABLE 7:** Linear regression results of perceived behavioral control scores.

Independent variables	Unstandardized coefficient		Standardized coefficient	*t*	*p*
	*B*	Standard error	Beta		
Constant	67.098	1.894		35.429	< 0.001
Household registration type	−5.401	0.915	−0.282	−5.904	< 0.001
Monthly household income	1.140	0.389	0.140	2.933	0.004

*Note: R* = 0.336; *R* ^2^ = 0.113.

The correlation between dimensions of the painless delivery intention self‐rating scale based on TPB with correlation coefficients of 0.536, 0.503, 0.533, 0.795, 0.870, and 0.799, respectively (*p* < 0.01), and the results are shown in Table 8. Among these, perceived behavioral control showed the strongest correlation with behavioral attitude (*r* = 0.870), which is the most direct predictor of behavioral intention in the TPB, making perceived behavioral control the strongest construct predicting behavioral intention toward painless childbirth.

**TABLE 8:** Results of correlation analysis between dimensions of the scale.

Dimension	Behavioral attitude (B)	Objective norm (C)	Perceived behavioral control (D)
Behavioral attitude(B)	1	0.795^∗∗^	0.870^∗∗^
Objective norm(C)		1	0.799^∗∗^
Perceived behavioral control(D)			1

*Note:* Basic knowledge​ (A).

^∗∗^At the 0.01 level (double‐tailed), the correlation is significant.

## 4. Discussion

### 4.1. Analysis of the Basic Information of the Respondents

In this study, 404 pregnant women who met the inclusion criteria for pregnancy testing in a tertiary hospital were investigated, which laid the foundation for the good development of this study. Among the surveyed pregnant women, the proportion of pregnant women aged 26–30 (38.86%) was the largest, which was in line with the optimal childbearing age [8], and the proportion of 30–35 years old (38.12%) was also higher, and the childbearing age was delayed. According to the survey on the participation of pregnant women in school courses, 74.01% of pregnant women have never participated in school courses for pregnant women, which has a lot to do with the requirements of epidemic prevention and control in 2020 to reduce the travel rate of citizens, and the implementation rate of online classes for pregnant women is low. Studies have shown that schools for pregnant women [9] have a positive effect on promoting the popularization of labor analgesia knowledge and improving the rate of labor analgesia. Through the targeted classes of pregnant women in the third trimester of pregnancy, the understanding of labor analgesia can be strengthened, and the intention of painless delivery can be improved. Regarding the phenomenon of low usage rate of online classes, it is necessary to combine information platforms such as “Internet + one‐stop innovative service for motherhood” to set up a repeat reminder function, optimize the viewing process, and increase the usage rate of online classes.

### 4.2. Analysis of the Basic Knowledge Score of the Self‐Rating Scale of Painless Delivery Intention Based on the TPB

This study showed that most of the basic knowledge scores of the self‐rating scale of painless delivery intention based on the TPB were at a medium level, and only 7 (1.733%) had a full score, indicating that the surveyed pregnant women had a slight grasp of the knowledge related to painless delivery, but the comprehensiveness and depth were insufficient. The four items of the basic knowledge dimension investigated pregnant women from four aspects: understanding of the types of labor analgesia, indications of labor analgesia, operation‐related knowledge, and postoperation care, and summarized the mastery of pregnant women’s knowledge of painless delivery. In the univariate analysis, there were statistically significant differences in the scores of basic knowledge of painless childbirth among pregnant women with different rural/urban backgrounds, different family monthly incomes, and different education levels, and the scores of women living in urban areas were higher than those of women living in rural areas. The score of pregnant women with a monthly household income between 15,000 and 20,000 is greater than that of pregnant women with a monthly household income of less than 15,000, which may be related to the higher cost of labor analgesia and not being covered by medical insurance. The score of basic knowledge was negatively correlated with the level of education, which was inconsistent with the results of two surveys on the influencing factors of labor analgesia knowledge in Shanxi and Hebei [10, 11] and may be related to the fact that people with lower education level have relatively free time, have more time after each pregnancy test, and have more opportunities to receive publicity related to childbirth knowledge.

The question “I understand the different methods of labor analgesia” scored the lowest, with an average score of 3.322, indicating that the majority of pregnant women do not have a comprehensive understanding of the types of labor analgesia. Ying et al. [12] conducted a survey on the knowledge of labor analgesia for outpatient pregnant women, showing that 11.90% of the pregnant women were unclear about the analgesic methods, and 24.40% of the pregnant women said that they were unclear about the complications of labor analgesia. In the survey on the acquisition of labor analgesia knowledge, 16.07% of pregnant women said that they had never known about labor analgesia, and the ways for pregnant women to obtain labor analgesia from high to low were TV media networks, relatives and friends, pregnant women’s schools, and others. Based on this conclusion, we should strengthen the promotion of the school curriculum for pregnant women to ensure that more pregnant mothers understand the knowledge of painless delivery before labor and improve the delivery experience.

### 4.3. Analysis of the Behavioral Attitude Score of the Self‐Rating Scale of Painless Delivery Intention Based on the TPB

In this study, 54.703% of pregnant women showed a high degree of enthusiasm toward painless delivery, with 54.703% of pregnant women having attitudes greater than 30 out of 40, and the study showed that [13] pregnant women would have a spontaneous fear of childbirth when they thought of the unpredictable pain of childbirth, and in order to alleviate this fear, they would tend to choose painless delivery, which was consistent with the survey of attitudes toward painless delivery. An international study [14] showed that the willingness of women to choose epidural analgesia increased from 27.9% before the onset of uterine contractions to 48.2%, and if all the pregnant women included in this study chose postpartum pregnant women, the behavioral attitude score about painless delivery choice should be improved. The differences in the behavioral attitude scores of pregnant women with different household income and different household registration types may be due to the different consumption concepts and understanding of painless delivery among pregnant women with different household registration types and monthly household income. This is consistent with Zhao’s [15] study that cost is a key factor challenging the promotion of painless delivery.

The question with the highest score was “If I meet the conditions in the future, I will choose neuraxial anesthesia and analgesia,” with an average score of 3.955, indicating that in China, which is more conservative, pregnant women often have altruistic psychology [16] or consider consumption and hope to choose painless delivery when conditions permit. The question with the lowest score was “I am very concerned about which hospital can be painless,” with an average score of 3.542, which may be related to the gradual strengthening of the standardization of prenatal examinations, the relatively fixed selection of hospitals for pregnancy testing, and the fact that most hospitals can achieve labor analgesia.

### 4.4. Analysis of Subjective Normative Scores on the Self‐Rating Scale of Painless Delivery Intention Based on TPB

There were 2 items in this dimension, and 367 people (90.842%) scored more than 6 points (10 points), indicating that pregnant women, as social persons, would be affected by the attitude of those around them toward painless delivery.

The subjective normative scores of pregnant women with different rural/urban types and monthly household income were different, which may be related to the fact that pregnant women with higher monthly household income will be subject to more social pressure.

### 4.5. Perceptual Behavior Control Score Analysis of Painless Delivery Intention Self‐Rating Scale Based on TPB

This study showed that the perceptual behavioral control score of painless labor was concentrated between 51 and 70 points, with 275 people (68.069%), but only 33 people (8.168%) scored 80 points. The average score of this dimension was generally high, with an average score ranging from 3.757 to 4.196, which may be related to the fact that pregnant women in the survey population, as independent adults, were completely predictable and able to control their own behavior. The perceptual behavior control scores of pregnant women with different rural/urban types and monthly household income were different, which was related to the increase in consumer spending behavior stimulated by the increase in familiarity with the medical environment and the increase in personal disposable income [17].

The question with the highest score in the intention behavior of pregnant women was “I have the right to choose neuraxial anesthesia and analgesia,” with an average score of 4.196, which may be related to the fact that pregnant women themselves have their own fixed income and the family status of pregnant women in the new era. The question with the lowest score for pregnant women was “neuraxial anesthesia analgesia does not leave sequelae,” which may be related to the pain in the needle at the epidural puncture site known to pregnant women from various sources and the mixed conclusions of studies on the effects of painless delivery on mothers and babies.

### 4.6. Correlation Analysis Between the Dimensions of the Painless Delivery Intention Self‐Rating Scale Based on the TPB

This study found that there was a positive correlation between the four dimensions of basic knowledge, behavioral attitude, subjective norms, and perceptual behavior control, which confirmed that there was a mutual promotion between whether the knowledge was comprehensive or not, whether the attitude was positive, the support of important others, and the perceived level of behavioral control. There is a strong correlation between behavioral attitudes and perceptual behavioral control, which is consistent with the findings of Shalini and Puneet [18], that is, attitudes affect the occurrence of behaviors. With a correlation coefficient of 0.870, perceived behavioral control exhibited the strongest association with behavioral attitude, a core determinant of behavioral intention within TPB. Given that behavioral attitude serves as the most proximal antecedent to behavioral intention in the TPB, this robust correlational relationship collectively identifies perceived behavioral control as the strongest predictor of pregnant women’s behavioral intention toward painless childbirth.

### 4.7. Recommendations for Improving the Intention of Choosing a Pain‐Free Delivery

This study suggests that the basic knowledge score was low, with an average score of 3.725 points, indicating that the low utilization rate of labor analgesia in China may be closely related to the low level of awareness of pregnant women about their related knowledge, and targeted health education should be carried out to enable pregnant women to master the knowledge of painless delivery and form correct cognition and attitude, so as to improve the use rate of painless delivery. Studies have shown that [19] 90% of pregnant women in the United States and Western Europe search the internet for pregnancy‐related health information. However, 66% of women participants believe that the information they get from a website may be inaccurate or misleading. Pregnancy information is widely available online due to the widespread use of the internet, and it is important for healthcare professionals to monitor the quality and accuracy of the information patients receive from internet sources. We should take advantage of the opportunity of each pregnancy test to actively answer the doubts of pregnant women in the midwifery clinic; make use of the careful curriculum of the pregnant women’s school; provide accurate and effective health education; and use multimedia methods such as WeChat groups, official accounts, and Douyin to shorten the distance between midwives, obstetric nurses and pregnant women and popularize the knowledge of painless delivery. Therefore, in the future, we should pay full attention to the health education of perinatal women, strengthen the publicity of labor analgesia, and improve the rate of natural delivery, so as to change the current situation of high cesarean section rate in China.

#### 4.7.1. Health Education Needs to Be Strengthened

Health education is an important means of contemporary healthcare developments [20–27], guiding people to establish concepts of health and strengthen awareness through knowledge to change behavior [28–32]. Nowadays, a variety of forms of health education facilitate the popularization of knowledge [33, 34], such as lectures, health education brochures, personalized health education, quality control circles, and mind maps [35]. However, when it is applied to outpatient pregnant women, the education objects are not concentrated, and it has been inconvenient for pregnant women to participate in on‐site classes as a key group during the 2020‐2023 pandemic. In this study, the identified score of basic knowledge is low, thus suggesting intensive implementation of health education as an imperative, which can be conducive to pregnant women understanding painless delivery options and choosing the appropriate delivery method.

#### 4.7.2. Promote the Role of Schools for Pregnant Women in Promoting Natural Childbirth

The School for Pregnant Women [36] is a health education communication organization with a unified purpose and plan to disseminate information and knowledge about maternal healthcare to the audience and to promote maternal health. According to Lasswell’s 5 W transmission model, in the pregnant women’s school, the communicators are professional midwives, and the content is about painless delivery; the communication channels are mainly personal communication and mass communication, and the recipients are pregnant women and their families in the third trimester. Schools for pregnant women should formulate a thorough plan and set up compulsory completion courses according to the different gestational weeks of pregnant women, so as to improve the awareness rate of pregnant women about painless delivery and promote natural childbirth.

#### 4.7.3. Harness the Role of Midwifery Clinics in Promoting Natural Childbirth

Pregnancy is a natural physiological process, and unlike other diseases, midwives play an important role in the process of childbirth. Studies have shown that midwifery clinics [37] can reduce the rate of cesarean section and pain during delivery and can help pregnant women maintain normal weight gain and improve delivery outcomes. Midwifery clinics should be fully utilized to provide a continuum of care for pregnant women, establish long‐term interactions with pregnant women, facilitate continuous information support, focus on the needs of pregnant women, and help pregnant women build confidence in natural childbirth.

WHO [28, 29] recommends some nonclinical actions that can reduce medically unnecessary use of caesarean sections, within the overall context of high‐quality and respectful care: Educational interventions that engage women actively in planning for their birth, such as childbirth preparation workshops, relaxation programs, and psychosocial support where desired, for those with fear of pain or anxiety. Implementation of such initiatives should include ongoing monitoring and evaluation, use of evidence‐based clinical guidelines, performing regular audits of caesarean section practices in health facilities, providing timely feedback to health professionals about the findings, and requirement for a second medical opinion for a caesarean section decision in settings where this is possible.

For the sole purpose of reducing caesarean sections, some interventions have been piloted by some countries but require more rigorous research: A collaborative midwifery−obstetrician model of care, for which care is provided primarily by midwives, with 24‐h back‐up from a dedicated obstetrician and financial strategies that equalize the fees charged for vaginal births and caesarean sections.

### 4.8. Limitations

Due to the limitation of the actual scale of the survey, the design of this study only considered some of the demographic characteristics of pregnant women, such as the previous experience of anesthesia and analgesia. While not included in the study, other characteristics, the study could not fully reflect how all pregnant women perceive the development of painless delivery.

## 5. Conclusion

The results of this study suggest two main conclusions.

One, that there is an ongoing need to generate international evidence on the current status of painless delivery intentions among pregnant women and identify influencing factors.

Two, that pregnant women’s intention on alternative painless delivery needs additional health education efforts.

### 5.1. Implications for Practice

The identified score of basic knowledge amongst pregnant women is low, thus suggesting the need for intensive implementation of health education as an imperative strategy conducive to a better understanding of alternative painless delivery options. This evidence generated supports healthcare professionals when deciding the focus of their interventions.

## Author Contributions

Xue Cheng and Xiaoling Li: conceptualization, design, and initial manuscript writing. Jinlan Xu: methodology and data curation. Paulo Moreira: revision and editing of the final manuscript.

## Funding

This study was supported by the Shandong First Medical University (Shandong Academy of Medical Sciences) Youth Science Fund Cultivation and Funding Program “Construction and Application of Evaluation System for Pregnant Women’s Intention to Give Birth without Pain” (202202‐08).

## Ethics Statement

The study was approved by the Ethics Committee of the First Affiliated Hospital of Shandong First Medical University, approval number: 02‐20240,208.

## Consent

The authors have nothing to report. No patient data were used for this study.

## Conflicts of Interest

The authors declare no conflicts of interest.

## Data Availability

Data are available from the authors at reasonable request.
